# 剂量调整后的奥加伊妥珠单抗两剂疗法治疗复发/难治急性B淋巴细胞白血病的疗效分析

**DOI:** 10.3760/cma.j.issn.0253-2727.2023.11.005

**Published:** 2023-11

**Authors:** 丽红 安, 德峰 赵, 瑞峰 侯, 欢欢 关, 红 闫, 跃辉 林, 春容 童, 彤 吴, 双又 刘

**Affiliations:** 北京高博博仁医院，北京 100070 Beijing GoBroad Boren Hospital, Beijing 100070, China

**Keywords:** 白血病，B淋巴细胞，急性, 复发/难治, CD22, 奥加伊妥珠单抗, Leukemia, B-cell, acute, Relapsed/refractory, CD22, Inotuzumab ozogamicin

## Abstract

**目的:**

观察靶向CD22的抗体奥加伊妥珠单抗两剂疗法对于多线治疗后特别是嵌合抗原受体T细胞（CAR-T细胞）治疗后复发/难治急性B淋巴细胞白血病（R/R B-ALL）的疗效。

**方法:**

回顾性分析了2020年3月至2022年9月在北京高博博仁医院血液科接受两剂CD22单抗治疗并评估了疗效的R/R B-ALL患者（包括成人和儿童）。所有患者治疗前均经流式细胞术检测证实表达CD22抗原（>80％白血病细胞表达CD22）。所用CD22单抗为注射用奥加伊妥珠单抗，剂型为1 mg/瓶。成人每剂1 mg，儿童每剂不超过1 mg，最大剂量0.85 mg/m^2^；每例患者用两剂，总剂量均小于标准剂量1.8 mg/m^2^。

**结果:**

共纳入21例R/R B-ALL患者，5例儿童（<18岁）和16例成人。17例患者骨髓/外周血白血病细胞比例为5.0％～99.0％或伴有髓外病变，4例仅骨髓微小残留病（MRD）阳性。14例患者接受过CD19和CD22 CAR-T细胞治疗，4例接受过CD19 CAR-T细胞治疗，3例接受过CD3/CD19双特异性抗体治疗。11例为异基因造血干细胞移植后患者。经CD22单抗治疗后，14例（66.7％）患者获得完全缓解（CR，其中1例为MRD阳性CR），4例仅有骨髓MRD阳性者均转为MRD阴性。6例CD22 CAR-T细胞治疗失败者中，4例经随后的CD22单抗治疗达到CR。7例（33.3％）患者治疗无效。5例（23.8％）患者在抗体治疗过程中发生Ⅰ～Ⅲ级肝毒性，1例无效患儿在挽救性移植过程中发生肝静脉闭塞病（HVOD），经治疗后痊愈。

**结论:**

对于多线治疗（包括移植及CD19/CD22 CAR-T细胞）后的R/R B-ALL患者，两剂CD22抗体方案疗效好、费用低，肝毒性和HVOD发生率低。

## Abstract (en)

**Objective:**

To observe the treatment response of a two-dose regimen of inotuzumab ozogamicin（inotuzumab）, a monoclonal antibody targeting CD22, for patients with heavily treated relapsed/refractory B-cell acute lymphoblastic leukemia（R/R B-ALL）, including those failed or relapsed after chimeric antigen receptor（CAR）-T-cell therapy.

**Methods:**

Pediatric and adult patients who received two doses of inotuzumab and who were evaluated after inotuzumab treatment were included. Antibody infusions were performed between March 2020 and September 2022. All patients expressed CD22 antigen as detected by flow cytometry（>80％ leukemic cells displaying CD22）before treatment. For adults, the maximum dosage per administration was 1 mg（with a total of two administrations）. For children, the maximum dosage per administration was 0.85 mg/m^2^（no more than 1 mg/dose; total of two administrations）. The total dosage administered to each patient was less than the standard dosage of 1.8 mg/m^2^.

**Results:**

Twenty-one patients with R/R B-ALL were included, including five children（<18 years old）and sixteen adults. Seventeen patients presented with 5.0％–99.0％ leukemic blasts in the bone marrow/peripheral blood or with extramedullary disease, and four patients were minimal residual disease（MRD）-positive. Fourteen patients underwent both CD19 and CD22 CAR-T-cell therapy, four underwent CD19 CAR-T-cell therapy, and three underwent blinatumomab therapy. Eleven patients underwent allogeneic hematopoietic stem cell transplantation（allo-HSCT）. After inotuzumab treatment, 14 of 21 patients（66.7％）achieved a complete response（CR, one was MRD-positive CR）, and all four MRD-positive patients turned MRD-negative. Four of six patients who failed recent CD22 CAR-T-cell therapy achieved a CR after subsequent inotuzumab treatment. Seven patients（33.3％）demonstrated no response. Grade 1–3 hepatotoxicity occurred in five patients（23.8％）, one child with no response experienced hepatic veno-occlusive disease（HVOD）during salvage transplantation and recovered completely.

**Conclusion:**

For patients with heavily treated R/R B-ALL, including those who had undergone allo-HSCT and CD19/CD22 CAR-T-cell therapy, the two-dose regimen of inotuzumab resulted in a CR rate of 66.7％, and the frequency of hepatotoxicity and HVOD was low.

CD19或CD22特异性嵌合抗原受体（chimeric antigen receptor，CAR）T细胞疗法在复发/难治急性B淋巴细胞白血病（R/R B-ALL）中取得了令人振奋的结果[1]–[4]。然而，部分患者CAR-T细胞治疗后仍会复发，他们通常更难达到完全缓解（CR）。少数R/R B-ALL患者由于各种原因无法进行CAR-T细胞治疗，如严重感染或脏器损害，或无法采集淋巴细胞进行培养等。在这些情况下，靶向CD19或CD22的单克隆抗体（单抗）成为可选择的治疗方案。CD3/CD19双特异性抗体贝林妥欧单抗（blinatumomab）和CD22抗体偶联药物奥加伊妥珠单抗（inotuzumab ozogamicin）已被证实对R/R B-ALL治疗效果明显优于化疗[5]–[8]。针对CD19 CAR-T细胞或CD19抗体治疗后CD19抗原阴性者，CD22抗体治疗更具优势。

本研究中，我们观察了CD22抗体奥加伊妥珠单抗对已经接受过CD19/CD22 CAR-T细胞或CD19 抗体贝林妥欧单抗治疗后的R/R B-ALL患者的疗效。由于CD22抗体价格昂贵，为了节省成本，我们还根据药物剂型（1 mg/瓶）调整了每次给药剂量，并将每例患者标准剂量的三剂调整为仅给予两剂。

## 病例与方法

一、病例资料

本研究回顾性分析了2020年3月至2022年9月期间在本院血液科接受了两剂CD22单抗治疗并评估了疗效的R/R B-ALL患者（包括成人和儿童），缓解后患者随访至2023年1月15日。R/R B-ALL的诊断及疗效评估参照2020年NCCN临床实践指南第二版标准[9]。本研究获得了北京高博博仁医院伦理委员会的批准（批件号：KY2022-034-001）。患者或监护人均签署了CD22单抗用药知情同意。

治疗前所有患者均经流式细胞术（FCM）检测CD22抗原，>80％白血病细胞表达CD22抗原者始纳入治疗以保证靶向用药的合理性，所用仪器为BD公司的8色流式细胞仪（BD FACS Canto Ⅱ）。患者CD22的表达情况见图1。

**图1:**
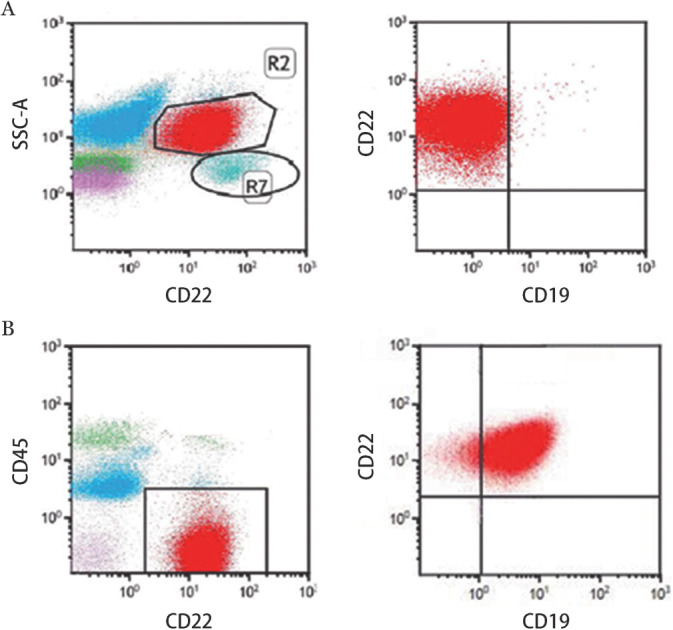
两例CAR-T细胞治疗后复发急性B淋巴细胞白血病患者流式细胞术检测CD22抗原表达情况（红色代表白血病细胞） A CD19阴性复发； B CD19阳性复发

二、CD22单抗用法

1. 两剂方案：所用CD22单抗为辉瑞公司生产的注射用奥加伊妥珠单抗（商品名：贝博萨，Besponsa），成人每剂最大剂量为1 mg（1 mg/瓶），儿童每次不超过1 mg，每剂最大剂量不超过0.85 mg/m^2^，每例患者用药2剂。

2. 配置及用法：先将药物溶于4 ml灭菌注射用水，再加入0.9％生理盐水50 ml，避光输注，液速50 ml/h。单抗输入前予以地塞米松5 mg及异丙嗪25 mg（儿童根据体重相应减量）预防过敏反应。

三、联合其他抗白血病用药

共9例患者抗体治疗期间短期使用了小剂量靶向药物及化疗药物。靶向药物包括维奈克拉（8例，10～200 mg/d）、酪氨酸激酶抑制剂（3例）及奥拉帕利（2例）等；化疗药物包括地塞米松（4例）、地西他滨（2例，10 mg/d）、长春地辛（3例，1～2 mg/次）及去甲氧柔红霉素（1例，5 mg，1次）。

四、疗效及不良反应评估

1. 疗效评估：完成第二剂CD22单抗输注后进行骨髓细胞形态学及微小残留病（MRD）检测。髓外病灶采用B超/CT/核磁/PET-CT检查评估疗效。

2. MRD检测：所有患者采用多参数FCM检测MRD，伴融合基因阳性患者同时采用实时定量PCR（RQ-PCR）（Applied Biosystems 7500，赛默飞世尔科技公司产品）检测融合基因。MRD阴性定义为FCM及 RQ-PCR检测值均<0.01％。

3. 不良反应评估：肝毒性分级采用2017年常见不良事件评价标准（CTCAE）5.0版。

## 结果

一、CD22单抗治疗前患者基本资料

患者临床情况详见表1。21例R/R B-ALL中，儿童（<18岁）5例，成人16例，中位年龄为29（4～72）岁。17例患者为血液学复发状态，其中16例形态学检查显示骨髓/外周血白血病细胞比例为5.0％～99.0％（3例伴有髓外病变），1例骨髓MRD阳性（FCM 1.75％）伴髓外病变。4例患者仅为MRD阳性，FCM显示骨髓肿瘤细胞0.05％～0.58％，其中1例伴E2A-PBX1融合基因阳性，定量PCR检测E2A-PBX1为0.7％。

**表1:** 21例复发/难治急性B淋巴细胞白血病患者CD22抗体治疗前临床特征

特征	例数	占比（％）
年龄（岁)		
儿童（<18)	5	23.8
成人	16	76.2
性别		
男	10	47.6
女	11	52.4
异基因造血干细胞移植		
移植后	11	52.4
未移植	10	47.6
CAR-T细胞治疗		
CD19 CAR-T细胞	4	19.0
CD19和CD22 CAR-T细胞	14	66.7
CD3/CD19抗体	3	14.3
疾病状态		
BM/PB肿瘤细胞（形态）5%～99%	16	76.2
BM MRD阳性（FCM）伴髓外病灶	1	4.8
BM MRD阳性（FCM，0.05%～0.58%）	4	19.0
伴不良遗传/分子学改变		
TP53基因突变	7	33.3
Ph-like	9	42.9
复杂核型	4	19.1
融合基因		
BCR-ABL	3	14.3
MLL-AF4	1	4.8
MEF2D-HNRNFUL1	1	4.8
E2A-PBX1	2	9.5

注 CAR-T细胞：嵌合抗原受体T细胞；BM：骨髓；PB：外周血；MRD：微小残留病；FCM：流式细胞术

除化疗外，14例患者接受过CD19和CD22 CAR-T细胞治疗，4例接受过CD19 CAR-T细胞治疗，3例接受过CD3/CD19双特异性抗体治疗。11例行异基因造血干细胞移植（allo-HSCT）。

所有患者肿瘤细胞均表达CD22抗原，CD22表达率为87.92％～100％。8例患者CD19抗原丢失，2例CD19抗原部分表达。

二、CD22单抗治疗

16例成人患者按1 mg/次输注，共2次。5例儿童患者输注剂量为：第1次0.54～0.85 mg/m^2^（总量0.6～1 mg），第2次0.54～0.84 mg/m^2^（总量0.5～1 mg）。每例患者用药总剂量均小于标准剂量1.8 mg/m^2^。间隔时间为7～15 d（有感染或其他并发症者第二剂输注时间后延）。详见表2。

**表2:** 21例复发/难治急性B淋巴细胞白血病患者CD22抗体治疗及疗效、不良反应

例号	年龄(岁)	体表面积(m^2^)	BM/PB肿瘤细胞比例（%）	髓外病灶	两次剂量	疗效	不良反应
1	28	1.57	65		1 mg×2	NR	肛周感染
2	7	0.89	0.25(BM FCM)		0.75 mg×2	CR	无
3	4	0.72	94		0.6 mg；0.5 mg	NR	肝静脉闭塞病
4	30	1.77	62.5		1 mg×2	CR	轻微头晕、恶心
5	29	1.82	5		1 mg×2	CR	无
6	41	1.50	99	多发	1 mg×2	NR	无
7	13	1.85	64		1 mg×2	NR	无
8	49	1.57	16		1 mg×2	CR	药物性发热、药物性肝损伤、轻度皮肤和肝脏GVHD
9	22	1.54	43		1 mg×2	NR	面瘫、药物性发热
10	12	1.17	28		1 mg；0.8 mg	CR	肠道感染(屎肠球菌)
11	6	0.87	87.5	双肾	0.7 mg；0.5 mg	CR	烦躁
12	19	1.70	27.9		1 mg×2	CR	无
13	20	1.94	6		1 mg×2	CR	药物性肝损伤
14	31	1.40	0.26(BM FCM)		1 mg×2	CR	药物性肝损伤
15	54	1.65	20		1 mg×2	CR	无
16	67	1.82	77		1 mg×2	NR	无
17	26	1.40	10	右侧咽隐窝	1 mg×2	CR	药物性肝损伤
18	37	1.54	1.75(BM FCM)	多发	1 mg×2	NR	无
19	54	1.64	0.05(BM FCM)		1 mg×2	CR	脓毒血症(大肠埃希菌)、药物性肝损伤
20	61	1.83	13.4		1 mg×2	CR	无
21	72	1.60	0.58(BM FCM)		1 mg×2	CR	无

注 BM/PB：骨髓/外周血；FCM：流式细胞术；CR：完全缓解；NR：未缓解；GVHD：移植物抗宿主病

三、疗效

第二剂CD22单抗输注后第4～14天进行疗效评估。21例患者中有14例（66.7％）获得CR，包括CR伴不完全血液学恢复（CR with incomplete blood count recovery，CRi），其中1例为MRD阳性CR；4例仅有骨髓MRD阳性患者均达到MRD阴性。分组疗效评估显示血液学复发患者CR率为58.8％（10/17），而MRD阳性患者均达CR（4/4）。尤为突出的是，6例复发后CD22 CAR-T细胞治疗失败患者中，4例经随后的CD22单抗治疗后达到CR。7例（33.3％）患者抗体治疗无效。详见表2。

在14例CR患者中，7例桥接allo-HSCT（2例为二次移植），其中4例（57.1％）持续CR 2～15个月，2例复发，1例发生移植后淋巴增殖性疾病。而在7例未移植的患者中，5例在CD22单抗治疗后3～6个月内复发，即使随后予以巩固治疗（靶向药物、抗体、局部放疗等）（图2）。

**图2:**
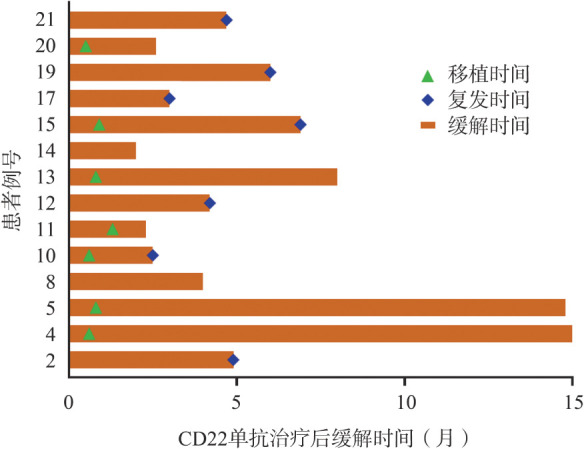
14例CD22抗体治疗后完全缓解患者的缓解时间

四、CD22单抗不良反应

抗体治疗期间出现的主要不良反应为肝损伤和发热。发生药物性肝损伤5例，表现为肝酶增高，无黄疸。丙氨酸转氨酶（ALT）42.6～584.8 U/L、天冬氨酸转氨酶（AST）41.7～349.0 U/L，其中1例合并排异因素（表现为用药前伴皮疹，用药后皮疹范围增大），均经保肝或联合激素治疗后好转。5例出现发热，其中感染性发热3例（分别为大肠埃希菌脓毒血症、屎肠球菌肠道感染及肛周感染），经抗感染治疗后好转；药物性发热2例，予以地塞米松、退热药后体温正常。2例出现中枢神经系统症状，其中1例面瘫，头颅CT平扫及疱疹病毒筛查未见异常；1例烦躁，均经激素治疗后好转。1例出现轻微头晕及恶心。

10例患者无任何不适。

此外，1例治疗无效的儿童患者挽救性移植后第19天（第1次单抗后47 d）出现肝静脉闭塞病（HVOD），肝功能异常最高值分别为ALT 656.8 U/L、AST 830.5 U/L、总胆红素192.06 µmol/L、直接胆红素98.22 µmol/L、间接胆红素43.84 µmol/L，予以去纤苷、甲泼尼龙、环孢素、芦可替尼、保肝等治疗后痊愈。

分析CD22单抗治疗前后的血常规变化，治疗前仅有1例MRD阳性患者血常规完全正常。绝大部分患者的白细胞、中性粒细胞及血红蛋白治疗前后变化不大，而1/3的患者治疗后血小板明显下降。

## 讨论

随着对B-ALL生物学特性的深入研究及治疗方案的改进，R/R B-ALL的疗效和预后得到很大改善。目前在R/R B-ALL中取得突破性进展的治疗是针对B细胞表面抗原CD19和CD22实施的CAR-T细胞免疫治疗和抗体免疫治疗[1]–[8]。CD19及CD22 CAR-T细胞治疗已在国内开展多年，但CD19和CD22抗体治疗由于之前药物的不可及性，国内应用较少。我院是国内最早开展CAR-T细胞临床研究的中心之一，面对CAR-T细胞治疗后复发的患者（特别是CD19阴性复发），或者因各种原因无法行CAR-T细胞治疗者，我们尝试采用了CD22抗体治疗。由于CD22单抗价格昂贵，我们将其剂量和使用方式做了调整以减轻患者经济负担。该药为1 mg/瓶，第1周期使用标准剂量为总量1.8 mg/m^2^，分3次使用，每周1次，每次剂量分别为0.8、0.5、0.5 mg/m^2^。我们的方案为成人每次1瓶（1 mg），儿童每次不超过1瓶，最大剂量不超过0.85 mg/m^2^，每例患者用药2次。

CD22抗体奥加伊妥珠单抗是一种抗体偶联药（ADC），由靶向CD22的人源化单抗和细胞毒性药物卡奇霉素组成。药物与肿瘤细胞表面CD22抗原结合后迅速内化，卡奇霉素在溶酶体中被释放出来，导致细胞DNA双链断裂和细胞凋亡，从而起到杀伤肿瘤细胞的作用[7],[10]–[11]。多个研究报道CD22单抗治疗R/R B-ALL的CR/CRi率为58.3％～80.0％[7]–[8],[10]–[11]。

本研究中，我们的患者人群为多线治疗（包括移植和CAR-T细胞）后复发的患者，85.7％（18/21）的患者接受过CD19 CAR-T细胞治疗（其中14例还接受过CD22 CAR-T细胞治疗）。11例进行过allo-HSCT。尽管如此，我们设计的两剂CD22单抗治疗方案可使患者的CR率达到 66.7％（14/21）。进一步分析发现，7例治疗无效者中6例为高肿瘤负荷（肿瘤细胞比例>50％或伴多发髓外病灶），而10例达到CR的血液学复发患者中仅有2例为高肿瘤负荷，4例MRD阳性患者疗效更佳，MRD均转阴（100％）；这些数据表明低肿瘤负荷患者对CD22抗体治疗的反应更好，提示R/R B-ALL患者一旦其他治疗无效可以尽早使用CD22单抗。此外，即使CD22 CAR-T细胞治疗失败的患者，换用CD22单抗仍有机会达到CR，本组6例CD22 CAR-T细胞治疗失败者中，4例经随后的CD22单抗治疗后获得CR。这6例CD22 CAR-T细胞治疗无效患者之前均接受过CD19 CAR-T细胞治疗，2例还接受过CD22 CAR-T细胞治疗，再次CD22 CAR-T细胞治疗失败的原因可能是免疫逃逸或产生了抗CAR的抗体[12]–[13]。而CD22单抗为ADC药物，和CAR-T的作用机制不同，主要依靠自带的细胞毒药物杀伤肿瘤细胞。

不良反应方面，文献报道肝毒性发生率为6.7％～50％[7]–[8],[10]–[11]，本研究中5例患者出现肝毒性，发生率为23.8％（5/21）。此外，CD22抗体治疗可以引起比较严重的HVOD，移植后患者发生率高于未移植患者，可高达22.8％～25.9％[7]–[8]。本组患者中所有患者抗体治疗期间无HVOD发生，1例治疗无效的儿童行挽救性移植后第19天出现HVOD，经积极治疗后好转。HVOD发病率占所有患者的4.8％（1/21），移植后患者的12.5％（1/8）。1例出现面瘫的患者由于疾病进展，不能排除肿瘤导致。无治疗相关性死亡。

奥加伊妥珠单抗安全性良好，儿童及老人均能耐受[10]–[11],[14]。本研究中3例60岁以上老人（最大72岁）治疗期间无不适，5例儿童患者中4例虽然单次剂量略超过标准剂量，也未显示更多不适。除单独使用外，奥加伊妥珠单抗还可以和低强度化疗联合使用以加强疗效[14]–[15]，本组患者中9例患者加了低剂量的靶向药及化疗药，耐受性仍然较好。

14例CR患者中的7例随后桥接了allo-HSCT，4例（57.1％）持续CR 2～15个月；而7例CR后未移植的患者中，5例在3～6个月内复发，即使随后给予了其他巩固治疗（由于CD22抗体高昂的价格，患者未能继续使用CD22抗体巩固治疗）；提示这些已经多线治疗后患者CR后行allo-HSCT有望获得较长时间的CR。

总之，对于多线治疗后包括移植和CAR-T细胞后的R/R B-ALL患者，CD22单抗仍可使2/3的患者获得CR。为了节省费用，我们基于剂型进行剂量调整后的两剂方案临床应用显示疗效和安全性良好，对中国及其他经济不发达地区患者非常有意义。

## References

[1] Maude SL, Laetsch TW, Buechner J (2018). Tisagenlecleucel in children and young adults with B-cell lymphoblastic leukemia[J]. N Engl J Med.

[2] Park JH, Rivière I, Gonen M (2018). Long-term follow-up of CD19 CAR therapy in acute lymphoblastic leukemia[J]. N Engl J Med.

[3] Fry TJ, Shah NN, Orentas RJ (2018). CD22-targeted CAR T cells induce remission in B-ALL that is naive or resistant to CD19-targeted CAR immunotherapy[J]. Nat Med.

[4] Liu S, Deng B, Yin Z (2021). Combination of CD19 and CD22 CAR-T cell therapy in relapsed B-cell acute lymphoblastic leukemia after allogeneic transplantation[J]. Am J Hematol.

[5] Kantarjian H, Stein A, Gökbuget N (2017). Blinatumomab versus chemotherapy for advanced acute lymphoblastic Leukemia[J]. N Engl J Med.

[6] Locatelli F, Zugmaier G, Rizzari C (2021). Effect of blinatumomab vs chemotherapy on event-free survival among children with high-risk first-relapse B-cell acute lymphoblastic leukemia: a randomized clinical trial[J]. JAMA.

[7] Kantarjian HM, DeAngelo DJ, Stelljes M (2019). Inotuzumab ozogamicin versus standard of care in relapsed or refractory acute lymphoblastic leukemia: Final report and long-term survival follow-up from the randomized, phase 3 INO-VATE study[J]. Cancer.

[8] DeAngelo DJ, Advani AS, Marks DI (2020). Inotuzumab ozogamicin for relapsed/refractory acute lymphoblastic leukemia: outcomes by disease burden[J]. Blood Cancer J.

[9] National Comprehensive Cancer Network (2020). NCCN Clinical Practice Guidelines in Oncology: Acute Lympho-blastic Leukemia. Version 2.

[10] Brivio E, Locatelli F, Lopez-Yurda M (2021). A phase 1 study of inotuzumab ozogamicin in pediatric relapsed/refractory acute lymphoblastic leukemia (ITCC-059 study)[J]. Blood.

[11] O'Brien MM, Ji L, Shah NN (2022). Phase II Trial of Inotuzumab Ozogamicin in Children and Adolescents With Relapsed or Refractory B-Cell Acute Lymphoblastic Leukemia: Children's Oncology Group Protocol AALL 1621[J]. J Clin Oncol.

[12] Rasche L, Vago L, Mutis T, Kröger N, Gribben J, Chabannon C (2022). Tumour Escape from CAR-T Cell. The EBMT/EHA CAR-T Cell Handbook[Internet].

[13] An L, Lin Y, Deng B (2022). Humanized CD19 CAR-T cells in relapsed/refractory B-ALL patients who relapsed after or failed murine CD19 CAR-T therapy[J]. BMC Cancer.

[14] Kantarjian H, Ravandi F, Short NJ (2018). Inotuzumab ozogamicin in combination with low-intensity chemo-therapy for older patients with Philadelphia chromosome-negative acute lymphoblastic leukaemia: a single-arm, phase 2 study[J]. Lancet Oncol.

[15] Jabbour E, Ravandi F, Kebriaei P (2018). Salvage Chemoimmunotherapy With Inotuzumab Ozogamicin Combined With Mini-Hyper-CVD for Patients With Relapsed or Refractory Philadelphia Chromosome-Negative Acute Lymphoblastic Leukemia: A Phase 2 Clinical Trial[J]. JAMA Oncol.

